# Selenomethionine Attenuated H_2_O_2_-Induced Oxidative Stress and Apoptosis by Nrf2 in Chicken Liver Cells

**DOI:** 10.3390/antiox12091685

**Published:** 2023-08-29

**Authors:** Lingyu Xie, Yibin Xu, Xiaoqing Ding, Kaixuan Li, Shuang Liang, Danlei Li, Yongxia Wang, Aikun Fu, Weixiang Yu, Xiuan Zhan

**Affiliations:** 1 Ministry of Agriculture and Key Laboratory of Animal Feed and Nutrition of Zhejiang Province, Key Laboratory of Animal Nutrition and Feed in East China, Feed Science Institute, College of Animal Science, Zhejiang University, Hangzhou 310058, China; 22117016@zju.edu.cn (L.X.); 21817002@zju.edu.cn (Y.X.); 12217006@zju.edu.cn (X.D.); 11517020@zju.edu.cn (K.L.); 22117026@zju.edu.cn (S.L.); 22117075@zju.edu.cn (D.L.); aikunfu@zju.edu.cn (A.F.); 2 Key Laboratory of Applied Technology on Green-Eco-Healthy Animal Husbandry of Zhejiang Province, College of Animal Science and Technology, College of Veterinary Medicine, Zhejiang A & F University, Hangzhou 311300, China; 20110046@zafu.edu.cn; 3 Animal Husbandry and Veterinary Services Center of Haiyan, Jiaxing 314300, China

**Keywords:** selenomethionine, sodium selenite, antioxidants, glutathione peroxidase, Nrf2

## Abstract

Earlier studies have shown that selenomethionine (SM) supplements in broiler breeders had higher deposition in eggs, further reduced the mortality of chicken embryos, and exerted a stronger antioxidant ability in offspring than sodium selenite (SS). Since previous studies also confirmed that Se deposition in eggs was positively correlated with maternal supplementation, this study aimed to directly investigate the antioxidant activities and underlying mechanisms of SS and SM on the chicken hepatocellular carcinoma cell line (LMH). The cytotoxicity results showed that the safe concentration of SM was up to 1000 ng/mL, while SS was 100 ng/mL. In Se treatments, both SS and SM significantly elevated mRNA stability and the protein synthesis rate of glutathione peroxidase (GPx) and thioredoxin reductase (TrxR), two Se-containing antioxidant enzymes. Furthermore, SM exerted protective effects in the H_2_O_2_-induced oxidant stress model by reducing free radicals (including ROS, MDA, and NO) and elevating the activities of antioxidative enzymes, which performed better than SS. Furthermore, the results showed that cotreatment with SM significantly induced apoptosis induced by H_2_O_2_ on elevating the content of Bcl-2 and decreasing caspase-3. Moreover, investigations of the mechanism revealed that SM might exert antioxidant effects on H_2_O_2_-induced LMHs by activating the Nrf2 pathway and enhancing the activities of major antioxidant selenoenzymes downstream. These findings provide evidence for the effectiveness of SM on ameliorating H_2_O_2_-induced oxidative impairment and suggest SM has the potential to be used in the prevention or adjuvant treatment of oxidative-related impairment in poultry feeds.

## 1. Introduction

A variety of stresses are connected to commercial poultry production, and most of them are linked with the excess production of free radicals and oxidative stress [1]. The mortality of chicken embryos is also associated with oxidative stress, which has a direct impact on economic benefits [2]. In the final stages of hatching, an embryonic chicken undergoes a transition to lung respiration, which results in a 60% increase in oxidative metabolism [2]. Due to the high proportion of highly polyunsaturated fatty acids, chicken embryos are sensitive to lipid peroxidation [3], which induces excessive free radicals and further causes oxidative stress [2]. Compared with other organs, the liver is more sensitive to oxidative damage [4]. It is the primary detoxifying and anabolic organ that excessively accumulates fats, metabolic products, and toxins, inducing free radicals and reactive oxygen species (ROS) [5]. To maintain a redox state, antioxidants are required to protect the liver from ROS damage.

Selenium is an essential microelement that is metabolized in the liver and generates major antioxidant selenoenzymes like glutathione peroxidase (GPx) and thioredoxin reductase (TrxR), which effectively improve antioxidant ability [6]. GPx is a key intracellular antioxidant enzyme that performs a primary protective role against cell membrane lipid oxidation and eliminates free radicals [7]. Selenocysteine (Sec), a major metabolite of selenium, is a vital part of the GPx active center [8]. Within the cytoplasm, the fully dissociated Sec can nucleophilically attack the polarized peroxyl bond and break it down to alcohol [7]. One study found that after changing Sec to cysteine in porcine phospholipid GPx, its antioxidant activity could decrease by 3000-fold [9]. TrxR is another selenoenzyme that contains a functional selenocysteine residue in the C-terminus [10]. TrxR can reduce oxidized thioredoxin, which serves as a strong electron and hydrogen donor to reduce oxidized peroxiredoxins in the reduced state and further eliminate lipid peroxides and H_2_O_2_ [7]. Furthermore, various studies have also reported that Se improves antioxidant ability [11,12] and reduces apoptosis [13] by activating the nuclear factor erythroid 2-related factor 2 (Nrf2), which is the key transcription factor regulating antioxidant enzyme genes including GPx and TrxR.

Currently, selenium sources used for feed supplementation are mainly categorized into inorganic forms of selenium (mainly sodium selenite, SS) and organic selenium (mainly Se-enriched yeast, SY). Compared with SS, organic Se shows less toxicity [14] and a greater bioavailability [6]. Se-enriched yeast (SY) is the most commonly used organic selenium supplementation [15]. A further study showed that more than 90% of Se in SY was in the form of selenomethionine (SM) [15], a methionine-derived compound replacing sulfur with selenium, which was also the predominant Se form in staple crops (e.g., corn, soybeans) [16]. In Se metabolism, SM replaces methionine incorporated into protein synthesis or is converted directly into Sec by the transsulfuration pathway. SS needs more steps to be reduced into selenide (H_2_Se) and finally converted into Sec [17]. Compared to SS, one study found that animals supplied with SM had a greater selenoenzyme activity and higher Se deposition [18]. According to our previous investigation, breeders who received an additional 0.15 mg/kg of SM exhibited the highest Se deposition in breeders, eggs, and offspring compared with the same dose of SS or SY [19]. Furthermore, compared with chelate selenium like Se-Gly, another kind of organic form that dissociates easily in vivo [20] and shows a lower absorption for its relatively weak coordinated bonds [21], SM was more stable and could be incorporated into protein synthesis to be maintained and utilized longer [18]. 

It is widely accepted that during embryonic development, the key factor influencing the formation of the antioxidant system is maternal nutrition [3]. Previous studies have suggested that maternal diet supplementation with Se from 0.1 to 0.5 mg /kg showed a positive association with Se contents found in both eggs and chicks [22,23,24]. Our study has further shown that compared with the basal diets of breeders, after adding 0.15 mg /kg SS or SY, the Se deposition in eggs was highest in SM intake [19]. Additionally, the breeding trial revealed that adding 0.15 mg/kg SM to the maternal diet resulted in a more notable decrease in chicken embryo mortality and hepatocyte apoptosis than SS. Moreover, it also exhibited superior antioxidant properties for the embryo liver to effectively combat oxidative stress induced by diquat (DIQ) [2]. To further explore the antioxidant effects of SS and SM on the chicken liver, this study evaluated the antioxidant effects by detecting cell viability, ROS generation, hepatocyte apoptosis, antioxidant ability, and the underlying mechanism based on the H_2_O_2_-stimulated chicken hepatocellular carcinoma cell line (LMH) to encourage further application in husbandry production.

## 2. Materials and Methods

### 2.1. Reagents and Chemicals

We obtained sodium selenite (SS) and selenomethionine (SM) from Sigma-Aldrich Chemical Co., Ltd. (St. Louis, MO, USA). Sangong Biotechnology Co., Ltd. (Shanghai, China) supplied the 30% H_2_O_2_. The 2′,7′-Dichlorodihydrofluorescein diacetate (DCFH-DA) used as a ROS detection probe was acquired from Aladdin Biochemical Technology Co., Ltd. (Shanghai, China).

### 2.2. Cell Culture

Professor Shiping Bai from the Animal Nutrition Research Institute at Sichuan Agricultural University generously provided the chicken hepatocellular carcinoma cell line (LMH). The cells were cultured in Dulbecco’s Modified Eagle Medium (Gibco, San Diego, CA, USA) supplemented with 10% fetal bovine serum FBS (Gibco, Invitrogen, Carlsbad, CA, USA) and 1% 100× penicillin–streptomycin (Solarbio, Beijing, China) at 37 °C with 5% CO_2_. 

### 2.3. Se cytotoxicity Assay

After the LMHs reached 80% confluence, they were transferred into a 96-well plate (1 × 10^4^). After overnight incubation, the cells were exposed to SS (sterile water dilution) or SM (diluted with 1% HCl) at concentrations of 0, 1, 10, 100, and 1000 ng/mL for 24 h. 

The cell proliferation assay was measured using the cell counting kit-8 method (Beyotime, Shanghai, China) as described in Section 2.4. 

### 2.4. Cell Viability Assay

After discarding the culture medium, 10 μL CCK-8 solution mixed with 100 μL fresh medium was injected into each well and incubated for 2 h at 37 °C. The value was detected at OD_450 nm_ (SpectraMax, Molecular Device Co., Sunnyvale, CA, USA). Finally, the cell viability ratio was obtained by calculating the values of treatment groups relative to the absorbance of the control group.

### 2.5. H_2_O_2_-Stimulated Oxidative Stress Model 

LMHs (1 × 10^4^) were placed in a 96-well plate and incubated overnight. After that, the cells were treated with 0, 50, 100, 150, 200, 500, 600, 700, 800, 900, and 1000 μmol/L of H_2_O_2_ (diluted with distilled water) for 24 h, respectively. After the cell viability assay described in Section 2.4 and LDH activity detection described in Section 2.6, 1000 μmol/L H_2_O_2_ was adapted for constructing the oxidative stress model. For H_2_O_2_ co-treatment, the overnight cultured cells were co-incubated with 1000 μmol/L H_2_O_2_ and 100 ng/mL SS or SM for 24 h. The cell proliferation assay was detected using the cell counting kit-8 method (Beyotime, Shanghai, China) described in Section 2.4. The detection of LDH activity is described in Section 2.6.

### 2.6. LDH Activity Assay

The LDH activity of the cell supernatant in the Se cytotoxicity assay and oxidative stress model was determined using an LDH Cytotoxicity kit (Jiancheng Bioengineering Institute, Nanjing, China). Briefly, after exposure to 1000 μmol/L H_2_O_2_ and with/without 100 ng/mL Se for 24 h, a combination of 120 μL cell-free supernatant and 60 μL LDH reaction mixture was incubated at room temperature in the dark for 30 min. The cell-free supernatant of cells without the H_2_O_2_ and Se treatments served as the negative control. The absorbance was detected at a wavelength of 490 nm.

### 2.7. Determination of Glutathione Peroxidase (GPx) and Thioredoxin Reductase (TrxR) mRNA Half-Lives

The mRNA half-life determinations were detected according to the method described previously with slight modifications [25]. The LMHs (1 × 10^6^) were placed in a 6-well plate. Following overnight incubation, LMHs were exposed to 100 ng/mL SS or SM for 24 h, with sterile water serving as the control. Then, 2 μg/mL actinomycin D (HY-17559, MedChemExpress, Monmouth Junction, NJ, USA) was added to the control and Se-treated cells. After incubating for 0, 1, 2, 4, 6, or 8 h, the RNA was extracted, and the mRNA level was subsequently assessed using real-time PCR, as will be discussed later.

### 2.8. Determination of GPx and TrxR Synthesis Rates

The protein synthesis rate determinations were assessed as described previously [26] with some modifications. LMHs (1 × 10^6^) were placed in a 6-well plate. Following overnight incubation, LMHs were exposed to 100 ng/mL SS or SM for 24 h, with sterile water serving as the control. Then, 10 μg/mL puromycin (AK058, GPCSCI, Beijing, China) was added to the control and Se-treated cells. After incubating for 0, 1, 2, 4, 6, or 8 h, the proteins were extracted and measured using a related ELISA Kit (Jiancheng Bioengineering Institute, Nanjing, China).

### 2.9. Measurement of Cellular Reactive Oxygen Species (ROS) 

The content of ROS was detected using the detection probe 2′,7′-dichlorodihydrofluorescein diacetate (DCFH-DA) from Beyotime, Shanghai, China. In brief, overnight-incubated cells were exposed to H_2_O_2_ alone or in combination for a duration of 24 h. After being harvested, the cells were rinsed with sterile PBS and stained with 20 μM DCFH-DA (dissolved in PBS) in a dark environment for 40 min at 37 °C. To examine the morphology of fluorescence, a fluorescence microscope from Micro-shot Technology Co., Ltd. in Guangzhou, China was utilized. Meanwhile, the measurement of fluorescence intensity was conducted at a wavelength of 488 nm/525 nm. To obtain the net fluorescence output, the fluorescence of PBS and cells without DCFH-DA staining were measured to account for background fluorescence.

### 2.10. Measurement of Oxidative and Antioxidative Indices

After being treated or co-treated with H_2_O_2_ for 24 h, cell supernatants were collected to detect the content of nitric oxide (NO) in the supernatants using a NO assay kit (Jiancheng Bioengineering Institute, Nanjing, China).

Additionally, after being washed with PBS, the harvested cells were lysed with 0.1% triton-X100. The cellular content of malondialdehyde (MDA) was detected using a microscale MDA assay kit (Jiancheng Bioengineering Institute, Nanjing, China). Furthermore, total antioxidant capacity (T-AOC) and total superoxide dismutase (T-SOD) were measured using a T-AOC assay kit and a T-SOD assay kit, respectively (Jiancheng Bioengineering Institute, Nanjing, China). The content of HO-1 and TrxR and the activity of GPx were detected according to the manufacturer’s instructions (Jiancheng Bioengineering Institute, Nanjing, China).

### 2.11. TdT-dUTP Terminal Nick-End Labeling (TUNEL) Assay

A TUNEL assay was used to measure DNA destruction in the final stages of apoptosis [27]. The overnight-cultured cells were treated or co-treated with H_2_O_2_ for 24 h. Untreated LMHs served as the control. Briefly, the cells were fixed in 4% formaldehyde for 15 min at room temperature and permeabilized with 1% Triton X-100 in PBS for 5 min. After washing with PBS, they were incubated with the TUNEL reaction mixture (Beyotime, Shanghai, China) for 1 h at 37 °C in the dark. Then, 4′,6-diamidino-2-phenylindole (DAPI) was used to identify the nuclei of LMHs. The samples were observed using a fluorescence microscope (MF52, Mshot, Guangzhou, China), and the green TUNEL-positive apoptotic cells were counted randomly.

### 2.12. Determination of Apoptosis-Related Indices

After different treatments, the cells were harvested and washed with PBS. The MMP was assessed using a JC-1 Mitochondrial Membrane Potential Assay Kit from Beyotime in Shanghai, China. The content of caspase-3 and Bcl-2 were detected using a caspase-3 assay kit and a Bcl-2 assay kit (Jiancheng Bioengineering Institute, Nanjing, China) referring to the instructions for the respective kits.

### 2.13. RNA Isolation and Quantitative Reverse Transcription PCR (qRT-PCR) Analysis

TRIzol reagent (Takara Biotechnology, Dalian, China) was used to extract the total RNA from the LMHs. Next, the RNA volume was optimized using nanodrop2000, and the integrity of the RNA was evaluated using 1% agarose gel electrophoresis. cDNA was synthesized using a HiScript ΙΙ1st Stand cDNA Synthesis Kit from Vazyme, located in Nanjing, China. Following that, ChamQ SYBR qPCR Master Mix (manufactured by Vazyme in Nanjing, China) was used for the amplification of the template cDNA (using 1 μL). Table 1 lists the used primers, with *gapdh* being considered as the gene for housekeeping. The qRT-PCR was observed using a CFX real-time PCR system (Bio-Rad, Foster, CA, USA) with the subsequent reaction conditions: denaturation at 95 °C for 30 s, 40 cycles for annealing, and extensions at 95 °C for 5 s and 60 °C for 30 s followed by a melting curve to analyze amplification specificity. Threshold cycle (Ct) values for target genes were adjusted to the Ct of the housekeeping gene and determined using the 2^−(ΔCt sample − ΔCt control)^ method [28].

### 2.14. Western Blotting Analysis

To lyse the LMHs (Beyotime, Shanghai, China), RIPA buffer was utilized, which consisted of a mixture of protease and phosphatase inhibitors. The extracted proteins were prepared in a standardized manner using the bicinchoninic acid (BCA) method (Jiancheng Bioengineering Institute, Nanjing, China). Equal amounts of total protein were combined with 5× DualColor Protein Loading Buffer (Fude Biological Technology Co., Ltd., Hangzhou, China) and heated at 99 °C for 5 min. Proteins were separated using 12% SDS-PAGE with 80 V for 20 min and 150 V for 40 min. Then, they were transferred to a nitrocellulose (NC) filter membrane in an ice–water mixture at 260 mA for 1 h. After 2 h of blocking in 5% skimmed milk at room temperature, it was incubated with primary antibodies (lddddd in Table 2) at 4 °C overnight. Following three washes with TBST, the secondary antibodies, HRP-conjugated goat anti-rabbit IgG (Abclonal, Wuhan, China), were incubated with the membrane at room temperature for 1.5 h. Imaging was achieved using the FDbio-Pico ECL kit (Fude Biological Technology Co., Ltd., Hangzhou, China). To standardize the expression of target proteins, β-actin served as the reference protein. Band intensities were quantified using ImageJ version 1.8.0 (Rasband, Bethesda, MD, USA).

### 2.15. Statistical Analysis

Each experiment was repeated at least three times individually. Results were presented as the mean ± standard deviation (SD) calculated using GraphPad Prism 9.0 (GraphPad Software, San Diego, CA, USA). Statistical comparisons (*p* < 0.05) between different groups were elevated using a two-sample unpaired Student t-test or one-way ANOVA with Tukey’s post hoc test using SPSS version 26.0 (SPSS Inc., Chicago, IL, USA).

## 3. Results and Discussion

### 3.1. Cytotoxicity of SM and SS on LMH Cells

**Figure 1:**
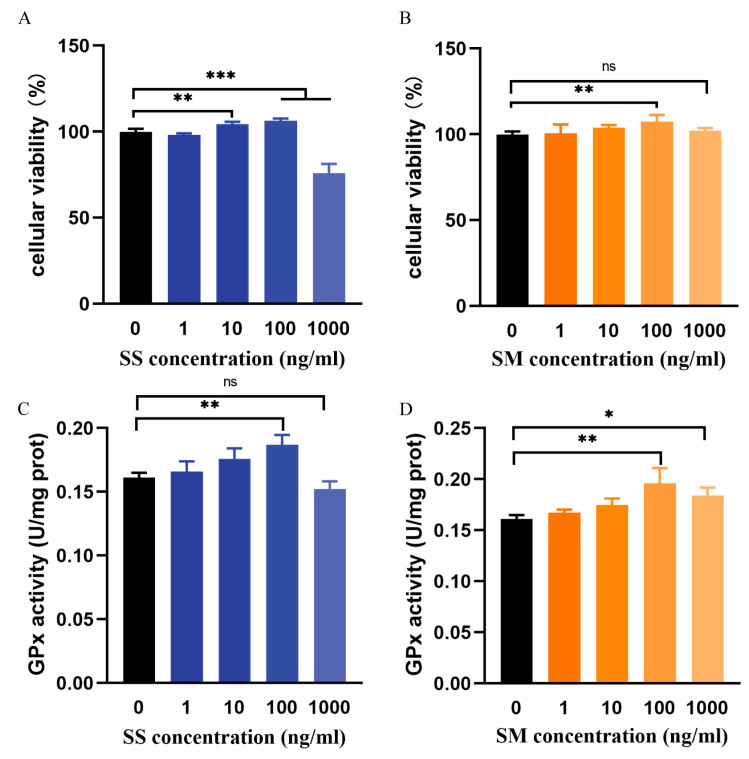
The cellular viability and GPx activity of LMHs under SS and SM treatment. LMHs were exposed to various concentrations (0, 1, 10, 100, and 1000 ng/mL) of SS or SM for 24 h. (**A**) SS-treated cell viability was measured using a CCK8 assay. (**B**) SM-treated cell viability. (**C**) GPx activity under the SS treatment. (**D**) GPx activity under the SM treatment. ns, no significant difference as compared to the control group; *, *p* < 0.05, **, *p* < 0.01, and ***, *p* < 0.001.

Although Se is an essential trace element for normal cell functions, high concentrations could induce toxic effects [29]. To test the cytotoxicity of SM and SS on LMH cells, the cells were exposed to varying concentrations (0, 1, 10, 100, and 1000 ng/mL) of SS or SM for 24 h. As shown in Figure 1, both 100 ng/mL SM and SS significantly increase the viability of LMH cells (*p* < 0.01), while the cells were significantly reduced under 1000 ng/mL SS treatment (*p* < 0.001), indicating that 1000 ng/mL SS was toxic for LMH cells. 

As Se serves as an important component of GPx [30], its activity was also detected. As shown in Figure 1B,C, both 100 ng/mL SS and SM significantly enhanced GPx activity (*p* < 0.01). Furthermore, 1000 ng/mL SM also increased the activity (*p* < 0.05), while SS did not increase the activity. A previous study using primary cultured chicken hepatocytes reported that the safe concentration of SM was up to 980.53 ng/mL, which also increased the activity of GPx compared with the control. However, 345.88 ng/mL SS markedly damaged the cell membrane, and the concentration that significantly increased GPx activity was found at 259.41 ng/mL [31]. Consistent with the previous study, our results also indicated a lower safe concentration for the SS treatment, as its cytotoxicity would lead to the inhibition of cell growth and a reduction in protein synthesis. [31]. Above all, these results indicated that SM had better effects on the regulation of cell viability and GPx activity. For the following studies, 100 ng/mL was chosen as the treatment concentration.

### 3.2. Effects of SS and SM on the mRNA Stability and Protein Synthesis of GPx and TrxR

The main biological form of Se is selenocysteine (Sec), which is co-translationally incorporated into selenoproteins and attribute functions [32]. In a growing peptide chain, Sec insertion combines the UGA stop codon with a specific stem-loop structure, the selenocysteine-insertion sequence (SECIS) element, and reprograms it to mean Sec instead of stop [32]. Since Se could incorporate into mRNA in the form of Sec and regulate the translation of selenoprotein, the mRNA half-life and protein synthesis rate of GPx and another Se-containing antioxidant enzyme, TrxR, under the Se treatments were detected to investigate the underlying effects.

**Figure 2:**
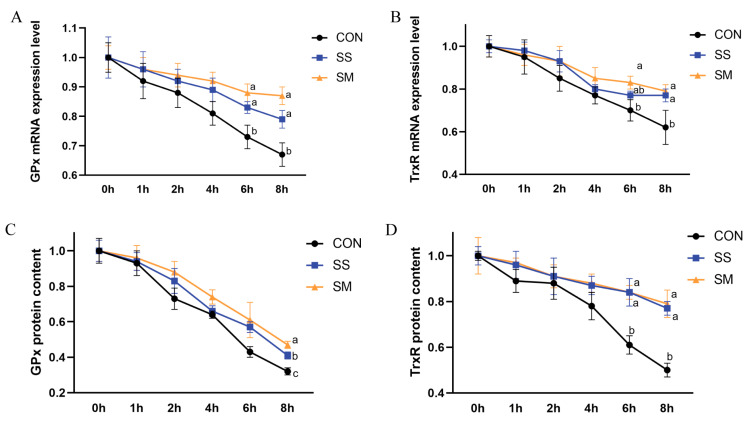
The effects of SS and SM on the mRNA stability and protein synthesis of GPx and TrxR. (**A**) The RNA half-life of GPx in the control, SS, or SM treatments at the time point after being treated with 2 μg/mL ActD. (**B**) The RNA half-life of TrxR in the control, SS, or SM treatments at the time point after being treated with 2 μg/mL ActD. (**C**) The protein synthesis rate of GPx in the control, SS, or SM treatments at the time point after being treated with 10 μg/mL puromycin. (**D**) The protein synthesis rate of GPx in the control, SS, or SM treatments at the time point after being treated with 10 μg/mL puromycin. Experimental data are shown as the mean ± SD. Each treatment was replicated three times. Different letters represented significant differences between treatments (*p* < 0.05).

The mRNA half-life was measured using actinomycin D, an RNA polymerase inhibitor, which stopped cell transcription and maintained it at 50% of the initial mRNA level, causing an mRNA decay curve [25]. In the presence of 100 ng/mL SS or SM, the mRNA levels of GPx were both higher than the control (*p* < 0.05) after the 6 h point (Figure 2A,B). For the mRNA levels of TrxR, the level of SM treatment was significantly higher than the control (*p* < 0.05) at the 6 h point, while both SS and SM enhanced the mRNA stability (*p* < 0.05) at the 8 h point. Li et al. found that 172.93 ng/mL SS treatment significantly elevated the mRNA half-life of selenophosphate synthetase in chicken embryo neurons [33]. Furthermore, 172.93 ng/mL SS was also found to elevate the mRNA half-life of selenoprotein W in chicken embryo neurons [25]. Additionally, Gallegos et al. reported that incubation with 345.86 ng/mL SS markedly increased the mRNA stability of thioredoxin reductase, a homodimeric Sec-containing protein, in HT-29 colon cancer cells [34]. Consistent with previous studies, our results indicated that both SS and SM could improve the mRNA stability of GPx and TrxR in LMHs.

Puromycin was used to measure protein synthesis, which was incorporated into the C terminus of nascent polypeptides and then translation elongation was halted [35]. As shown in Figure 2C,D, the GPx protein synthesis rate under SM treatment was the highest in the three groups. Additionally, compared with the control, the TrxR protein synthesis rates in both SS and SM treatments were higher (*p* < 0.05) after the 6 h point. A previous study also showed that 172.93 ng/mL SS treatment significantly increased the level and activity of thioredoxin reductase (TR) in MCF-7 breast cancer cells, HT-29 colon cancer cells, and A549 lung cancer cells [34]. The above results indicated that both SS and SM significantly elevated the protein synthesis rate, especially for SM in the GPx group.

### 3.3. Oxidative Stress Model of LMH Induced with H_2_O_2_

**Figure 3:**
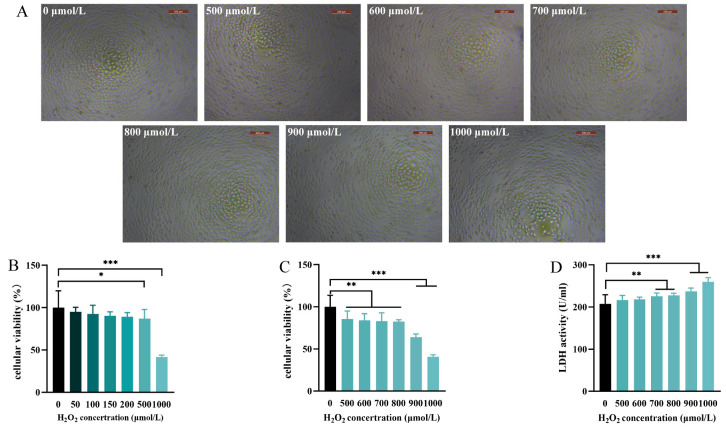
The effect of H_2_O_2_ on LMH proliferation. To construct an H_2_O_2_-stimulated oxidative stress model, LMHs were treated with H_2_O_2_ from 0 to 1000 ng/mL for 24 h. (**A**) The cellular morphology in each treatment was observed using a microscope with 100× magnification. (**B**,**C**) Cell viability was measured using a CCK8 assay. (**D**) The supernatant LDH activity of the H_2_O_2_ model. Experimental data are shown as mean ± SD. n = 6 replicates per treatment. *, *p* < 0.05; **, *p* < 0.01; ***, *p* < 0.001.

H_2_O_2_ is the most abundant ROS species, which is continuously generated during various physiological processes and diffuses freely into the interstitial areas surrounding cells in the liver [36]. It is a common inducer used to cause oxidative damage in vitro models [37]. Additionally, LDH is a stable cytosolic enzyme that appears extracellularly when the cell membrane is damaged [31]. Thus, H_2_O_2_-induced damage could be analyzed by measuring LDH activity in the culture supernatant. As shown in Figure 3B,C, the cellular viability of LMH was decreased significantly (*p* < 0.05) compared with the control when the concentration of H_2_O_2_ exceeded 500 μM, while viability was reduced by approximately 50% at the highest tested doses (1000 μM). Additionally, the supernatant LDH activity was increased (*p* < 0.05) when treated with 700 μM H_2_O_2_ and significantly increased up to 900 μM (*p* < 0.001) (Figure 3D). The untreated LMH cells maintained their normal structure and morphology, while a reduction in the cell number was induced from 500 to 1000 μM, and shrunken cells were observed in the 1000 μM treatment (Figure 3A). Thus, 1000 μmol/L H_2_O_2_ was selected to construct the oxidative stress model.

### 3.4. SS and SM Protect LMHs under Oxidative Stress

**Figure 4:**
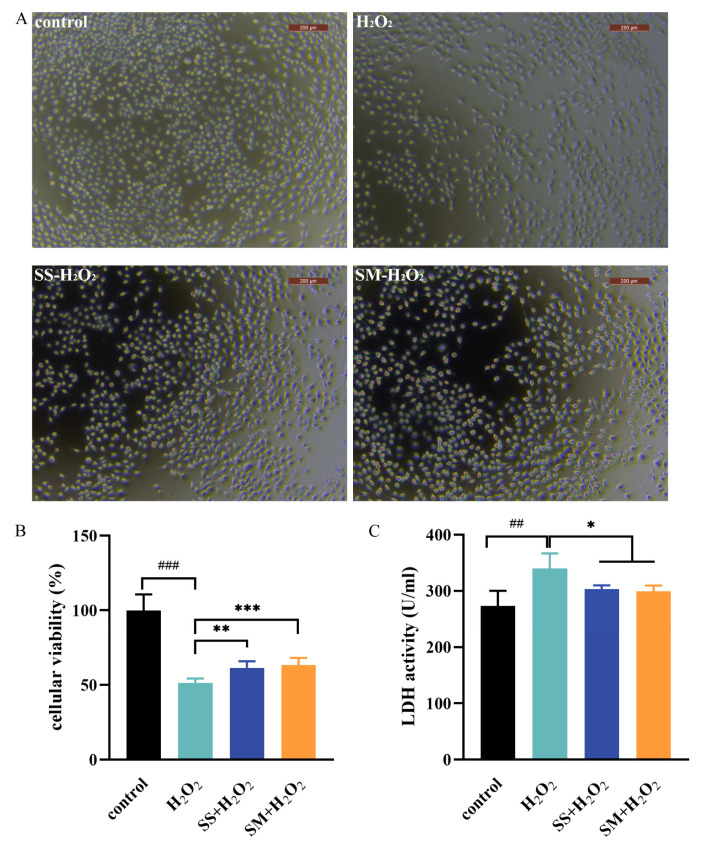
Effects of SS and SM on the cell viability and LDH activity in H_2_O_2_ co-treated LMHs. Cells were co-treated with 1000 μmol/L H_2_O_2_ and 100 ng/mL Se supplements for 24 h. (**A**) The representative cellular morphologies under the control and co-treatment were observed using a microscope with 100× magnification. (**B**) Cell viability was assessed using the CCK8 assay. (**C**) The supernatant LDH activity of the control and co-treatment. Experimental data are shown as mean ± SD. n = 6 replicates per treatment. ##, *p* < 0.01, and ###, *p* < 0.001 as compared with the control group; *, *p* < 0.05, **, *p* < 0.01, and ***, *p* < 0.001 as compared with the model group.

To investigate the effects of SM and SS on the LMHs under oxidative stress, cells were co-cultured with Se-supplements and H_2_O_2_ for 24 h. As shown in Figure 4, compared with the control group, the cellular viability of LMH in the model group was significantly decreased (*p* < 0.001), while the supernatant LDH activity increased significantly (*p* < 0.01). Compared with the model group, the cellular viability was elevated under both the SS-H_2_O_2_ (*p* < 0.01) and SM-H_2_O_2_ co-treatments (*p* < 0.001), and there was also a significant decrease in LDH activity (*p* < 0.05). Meanwhile, compared with the shrunken cells in the model group, the microscopy observation displayed normal structure and morphology in the SS-H_2_O_2_ and SM-H_2_O_2_ groups, which was similar to the control. The above results indicated the protective effects of SS and SM under oxidative stress. It was previously reported that feeding with 3 mg/kg Se had a healing effect on liver damage and enhanced the antioxidant ability to fight against chlorpyrifos-induced toxicity in rats [38]. Furthermore, another previous study also showed that Se-enriched food (total Se at around 29.2 µg/g) prevented mice liver tissues from severe damage induced by CCl4 [39].

### 3.5. Effects of SS and SM on the Oxidative and Antioxidative Parameters of LMHs under Oxidative Stress

**Figure 5:**
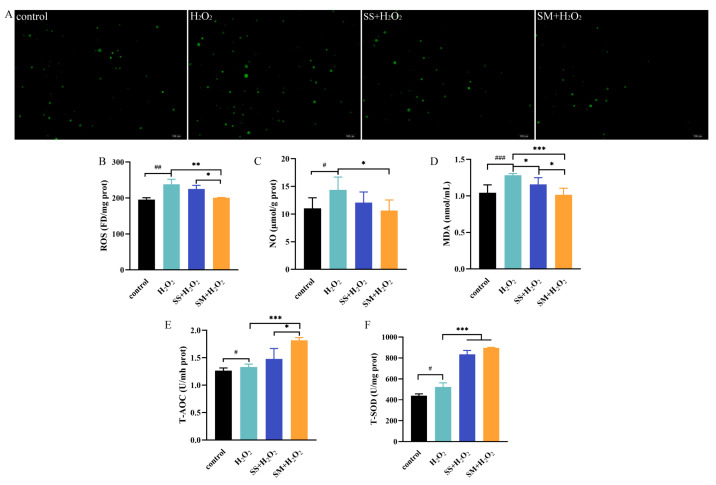
The protective effects of SS and SM on the oxidative stress in H_2_O_2_-co-treated LMHs. Cells were co-treated with 1000 μmol/L H_2_O_2_ and 100 ng/mL Se supplements for 24 h. (**A**) Epifluorescence images of reactive oxygen species (ROS) production in LMHs, which were monitored and photographed at 100× magnification. (**B**) Concentrations of ROS in LMHs under oxidative stress. n = 4 replicates per treatment. (**C**) Concentrations of nitric oxide (NO) in cellular supernatants under oxidative stress. n = 6 replicates per treatment. (**D**) The cellular content of malondialdehyde (MDA) under oxidative stress. n = 6 replicates per treatment. (**E**) The level of total antioxidation (T-AOC) capability in LMHs under oxidative stress. n = 6 replicates per treatment. (**F**) The activity of total superoxide dismutase (T-SOD) in LMHs under oxidative stress. n = 6 replicates per treatment. Experimental results are shown as the mean ± SD. #, *p* < 0.05, ##, *p* < 0.01, and ###, *p* < 0.001 as compared with the control group; *, *p* < 0.05, **, *p* < 0.01, and ***, *p* < 0.001 as compared with the model group.

Previous studies reported that H_2_O_2_ treatment significantly increased cellular ROS and downregulated the activities of antioxidative enzymes [40]. To determine the antioxidant effects of SS and SM, DCFH-DA was used as an indicator to detect hydroxyl, peroxyl, and other ROS in the treated LMHs. Compared with the control group, H_2_O_2_ treated for 24 h increased the positive cells and the content of cellular ROS markedly (*p* < 0.01) (Figure 5A,B). Compared with the model group, the stained cells in the SM-H_2_O_2_ treatment decreased, and the content of ROS also declined (*p* < 0.01). Two other biomarkers for oxidative damage including NO and MDA were also assessed. Compared with the model group, SS reduced H_2_O_2_-induced NO significantly (*p* < 0.05), while SM was found to inhibit the production of both NO (*p* < 0.05) and MDA (*p* < 0.001). Additionally, the cotreatment with H_2_O_2_-induced LMHs with SM markedly enhanced the level of T-AOC (*p* < 0.001) and increased the activity of T-SOD (*p* < 0.001) when compared to the model group, while SS only increased the activity of T-SOD (*p* < 0.001).

In a previous study, Wang et al. reported that a basal diet with 0.15 mg/kg Se protected the broiler liver from fluorine damage by increasing the activity of GPx and TrxR1 and reducing ROS production [41]. Moreover, the above effects in the SM treatment were better than SS [41]. In another study, 440 ng/kg Se in the basal diet was also found to alleviate hepatocyte structural damage, enhance GPx activity, and reduce MDA levels in the liver of geese experiencing oxidative stress [42]. Additionally, it was previously reported that maternal SS or SM supplementation for 8 weeks at 0.15 ppm markedly decreased ROS and MDA concentrations and increased GPx and T-SOD activities in the heat-stressed chick embryo. In addition, SM showed a higher efficacy than maternal SS [43]. Consistent with previous studies, the results of this study correlated with the proliferation of LMHs, indicating that SM exhibited effective antioxidant effects by reducing free radicals and elevating the activities of antioxidative enzymes, which performed better than SS.

### 3.6. Effects of SS and SM on the Cell Apoptosis of LMHs 

**Figure 6:**
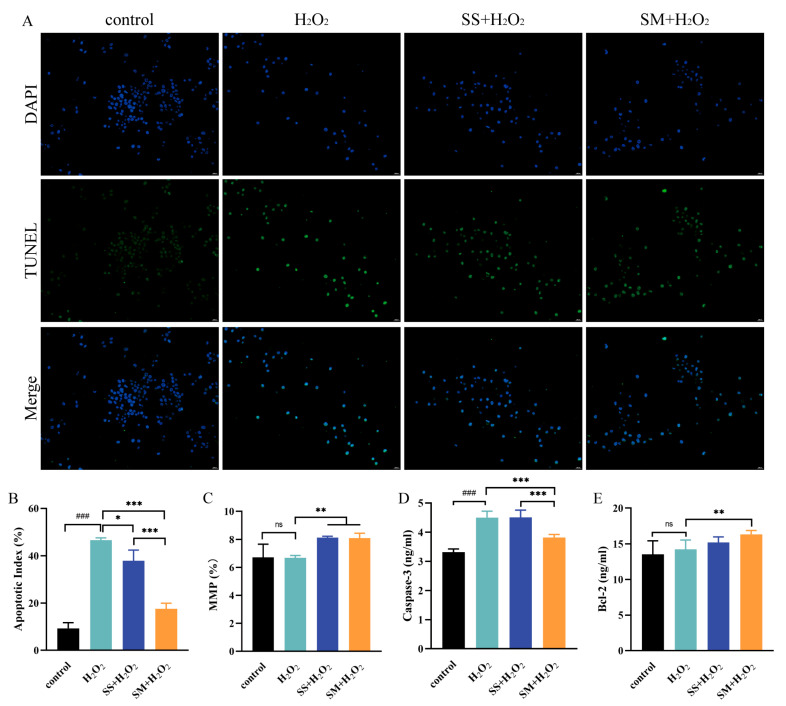
The effects of SS and SM on the apoptosis in H_2_O_2_-co-treated LMHs. (**A**) The nuclei stained with DAPI were blue, and the green apoptotic LMHs were stained with TUNEL. LMHs were monitored and photographed at 200× magnification. (**B**) Quantitative analysis of apoptotic cells in different treatments. Each treatment was photographed with three replicates randomly. (**C**) The mitochondrial membrane potential in different treatments. n = 3 replicates per treatment. (**D**) The content of caspase-3 in different treatments. n = 6 replicates per treatment. (**E**) The content of Bcl-2 in different treatments. n = 6 replicates per treatment. ###, *p* < 0.001, ns, no significant difference as compared to the control group; *, *p* < 0.05, **, *p* < 0.01, and ***, *p* < 0.001 as compared with the model group.

H_2_O_2_-induced oxidative stress would cause damage to DNA, lipids, and proteins, resulting in cell apoptosis and death [44,45]. TUNEL staining was used to investigate the effect of SS and SM on apoptosis. As shown in Figure 6A,B, there were no obvious apoptotic cells in the control group, while a considerable level of apoptosis was observed in the model group (*p* < 0.01). Moreover, the apoptotic indexes in the SS-H_2_O_2_ group (*p* < 0.05) and the SM-H_2_O_2_ group (*p* < 0.001) were significantly decreased compared with the control group. 

To further verify the role of SS and SM in H_2_O_2_-induced apoptosis, changes in the MMP and the level of caspase-3 were measured. Decreased MMP is crucial in the initial stage of apoptosis [46]. Conversely, both SS and SM elevated MMP compared with the model group (*p* < 0.01). Caspase-3 is a major execution protease in the apoptotic process, which degrades intracellular structural proteins and functional proteins, and ultimately induces cell death [47]. Compared with the control group, the level of caspase-3 increased significantly in the model group (*p* < 0.001), and then significantly decreased with SM-H_2_O_2_ co-treatment (*p* < 0.001). Furthermore, SM also increased the level of B cell lymphoma-2 (Bcl-2) under the H_2_O_2_ condition (*p* < 0.001), which is an important anti-apoptosis protein [48]. Se-related treatments have shown protective effects against apoptosis and tissue damage. A previous study reported that Se-enriched plant supplements (total Se content up to 1.43 g/kg) significantly decreased apoptosis of ovarian cells, downregulated the caspase-3 expression level, and upregulated the Bcl-2 expression level in aging laying hens [11]. Wang et al. found that 500 nM SS or 30 μM SM significantly inhibited apoptosis and restored the MMP level in mice liver cells. In that same study, 0.45 mg/kg dietary selenium also markedly improved hepatic injury in high-fat diet mice [49]. Additionally, earlier research has shown that overexpression of the chicken selenoprotein W gene prevented hepatic cells from apoptosis by upregulating the Bcl-2 expression level and downregulating the level of caspase-3 [50]. 

Consistent with previous studies, our results indicated that Se supplements can reduce apoptosis, especially for SM, by increasing the total antioxidant ability, decreasing the level of cellular ROS, and increasing Bcl-2 content while decreasing caspase-3 content.

### 3.7. Effects of SS and SM on the Nuclear Factor Erythroid 2-Related Factor 2 (Nrf2) and Downstream Regulators under Oxidative Stress

Nrf2 is an important regulator that controls the basal and induced expression of an array of antioxidant response genes to protect cells from oxidative damage [51]. The antioxidant defense systems of Nrf2 include the major Se-containing antioxidant enzymes GPx and TrxR, the stress response proteins, such as Herne oxygenase-1 (HO-1) [51]. 

**Figure 7:**
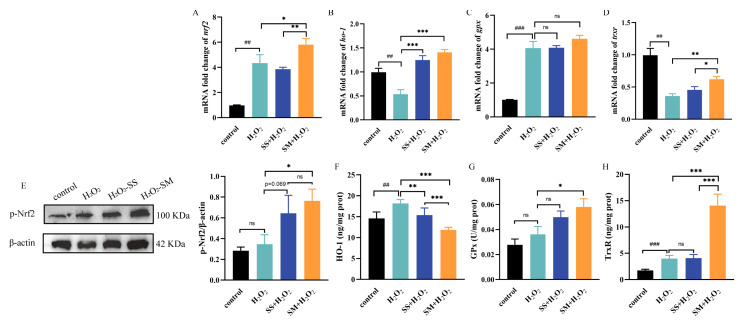
The effects of SS and SM on Nrf2 and downstream regulators under oxidative stress. (**A**) The gene expression of Nrf2 in the control, H_2_O_2_ treatment, and co-treatments. (**B**) The gene expression of HO-1 in the control, H_2_O_2_ treatment, and co-treatments. (**C**) The gene expression of GPx in the control, H_2_O_2_ treatment, and co-treatments. (**D**) The gene expression of TrxR in the control, H_2_O_2_ treatment, and co-treatments. (**E**) The protein expression of p-Nrf2 of LMHs in different treatments. (**F**) The protein level of HO-1 in different treatments. (**G**) The GPx activity in different treatments. (**H**) The protein level of TrxR in different treatments. Experimental data are shown as the mean ± SD. n = 3 replicates per treatment. ##, *p* < 0.01, ###, *p* < 0.001, ns, no significant difference as compared to the control group; *, *p* < 0.05, **, *p* < 0.01, and ***, *p* < 0.001 as compared with the model group.

To assess the effects of SS and SM on the Nrf2 pathway, the expression levels of Nrf2 and downstream genes were first detected. As shown in Figure 7, H_2_O_2_ treatment significantly upregulated the expression of Nrf2 (*p* < 0.01) and GPx (*p* < 0.001), while downregulated the expression of HO-1 (*p* < 0.01) and TrxR (*p* < 0.01). The disrupted gene expression of the Nrf2 pathway reflected the H_2_O_2_-induced damage to the LMHs, which could be an overall regulation to resist the damage. For SM-H_2_O_2_ cotreatment, the Nrf2 (*p* < 0.05), HO-1 (*p* < 0.001), and TrxR (*p* < 0.01) gene expressions were all upregulated compared with the H_2_O_2_ model group. Additionally, SS only upregulated the gene expressions of HO-1 (*p* < 0.001), suggesting a weaker regulation effect compared to SM.

The Western blot results indicated that both SS and SM co-treatments significantly increased the phosphorylation level of Nrf2 compared with the model group (*p* < 0.01). Furthermore, SM also markedly increased the activity of GPx (*p* < 0.05) and the level of TrxR (*p* < 0.001). However, the protein levels of HO-1 in both SS (*p* < 0.01) and SM (*p* < 0.001) co-treatments were significantly inhibited. Similar to our results, another study found that rosiglitazone (a PPARγ agonist) protected the central nervous system from oxidative damage by inhibiting ROS, elevating the functions of antioxidant enzymes, and concurrently reducing the protein expression of HO-1 [52]. In another study, Se treatments (0.5 mg/kg⋅BW) in rabbit brains damaged by cadmium (Cd) alleviated oxidant stress by increasing the activity of GPx and superoxide dismutase (SOD), while it also inhibited the HO-1 level [53].

Mateusz et al. reported that valproic acid, an anti-epileptic drug, did not affect the protein abundance of Nrf2 in treated murine embryonic fibroblasts, but it downregulated the HO-1 level for the elevated proteasomal degradation [54]. However, since the protein levels of Nrf2, GPx, and TrxR were markedly increased, the reason for proteasomal degradation was excluded. HO-1 is a double-edged sword in antioxidant defense. On the one hand, it degrades heme into potent scavengers for oxygen radicals; on the other hand, it also produces iron-dependent ROS and lipid peroxidation [55]. Generally, Bach1 as a repressor would inhibit the expression of HO-1, but only an adequate amount of heme could abrogate the repression of Bach1 to express HO-1 [56]. There is a feedback loop when ROS decreases, where Bach1 binds to the upstream of HO-1 to stop it from overexpressing [57]. In addition, several microRNAs were also found to inhibit the expression of HO-1 [56,58]. Regarding the discrepancy between the HO-1 mRNA and protein expression levels, Hung et al. found that the eukaryote translation initiation factors (eIFs) can be modified to inhibit the HO-1 translation [59]. In our results, the upregulated Nrf2 bound to the antioxidant response element (ARE) to promote the antioxidant genes, including HO-1, GPx, and TrxR [51]. Nevertheless, considering the notable increase in GPx and TrxR levels in the Se treatments, the levels of ROS, NO, and MDA were substantially reduced, similar to the control, especially for the SM treatments. Thus, we suppose the feedback loop would stop HO-1 overexpression in the translation way as former studies described. However, the underlying mechanisms need to be further investigated.

Compared with HO-1 exerting antioxidant effects indirectly, the “classical” antioxidant enzymes like GPx and TrxR display the real driving force of the antioxidant effects [55]. Zhao et al. reported that 0.3 mg/mL Se-enriched soy protein effectively protected H_2_O_2_-exposed Caco-2 cells from oxidative stress by activating the Nrf2 pathway and elevating the activities of GPx and SOD [60]. Additionally, 0.3 mg/kg SY supplement in broiler diets was found to alleviate liver damage-stimulated oxidative stress by activating the Nrf2 pathway and downstream GPx and quinone oxidoreductase 1 (NQO1) [61]. Furthermore, 0.1 μM biogenic nano-selenium also effectively activated the Nrf2 pathway and elevated GPx activity in porcine jejunum epithelial cells [62].

Above all, these results indicate that activated Nrf2 and downstream major antioxidant selenoenzymes might be responsible for protecting SM against H_2_O_2_-induced oxidative damage.

## 4. Conclusions

In this study, we first verified that SM is less toxic than SS. Meanwhile, both SS and SM significantly increased the contents of selenoenzymes with improved mRNA stability and protein synthesis rates. In the H_2_O_2_-induced oxidant stress model, SM markedly improved proliferation and reduced apoptosis of LMHs by reducing free radicals and elevating the activities of antioxidative enzymes, which performed better than SS. Overall, SM exerted stronger antioxidant effects by stimulating the Nrf2 pathway and further elevating the activities of key antioxidant selenoenzymes downstream. Taken together, the above results indicated that SM had better antioxidative effects than SS and provided an alternative viewpoint and method for the prevention and improvement of oxidative stress-related diseases in poultry.

## Figures and Tables

**Table 1:** The designed primers.

Gene	Accession Number	Forward Sequence (5′-3′)	Reverse Sequence (5′-3′)	Product Size
*Nrf2*	NM_205117.1	AGAAAACGCTGAACCACCAATC	GCTGGGTGGCTGAGTTTGATTA	217 bp
*TrxR*	NM_001030762.3	GGGGTCTTGGAGGAACATGTG	CCCCAGTTCAGTGAGCCAATG	187 bp
*GPx*	NM_001277853.2	CTGCAACCAATTCGGGCAC	CGCACTTCTCGAACATGGTG	116 bp
*HO-1*	NM_205344.1	TGTCCCTCCACGAGTTCAAGC	GACAGGTCTCCCAAATAGCGG	301 bp
*GAPDH*	NM_204305.1	CCTCTCTGGCAAAGTCCAAGTG	GGTCACGCTCCTGGAAGATAGT	176 bp

**Table 2:** The primary antibodies in this study.

Antibody Name	Dilution Ratio	Manufacturers
**β-actin**	1:10,000	Abclonal, Biotechnology Co., Ltd., Wuhan, China
**p-Nrf2**	1:1000	HuaAn Biotechnology Co., Ltd., Hangzhou, China

## Data Availability

Data will be provided on reasonable request.
